# Two Intra-Individual ITS1 rDNA Sequence Variants Identified in the Female and Male *Rotylenchulus reniformis* Populations of Alabama

**DOI:** 10.3390/plants13010005

**Published:** 2023-12-19

**Authors:** Seloame T. Nyaku, Sowndarya Karapareddy, Ernst Cebert, Kathy Lawrence, John S. Y. Eleblu, Govind C. Sharma, Venkateswara R. Sripathi

**Affiliations:** 1 Department of Crop Science, College of Basic and Applied Sciences, University of Ghana, Legon, Accra P.O. Box LG 44, Ghana; seloame.nyaku@gmail.com; 2 Department of Biological and Environmental Sciences, Alabama A&M University, Huntsville, AL 35811, USAgovind.sharma@aamu.edu (G.C.S.); 3 Department of Entomology and Plant Pathology, Auburn University, Auburn, AL 36849, USA; lawrekk@auburn.edu; 4 West Africa Centre for Crop Improvement, College of Basic and Applied Sciences, University of Ghana, Legon, Accra P.O. Box LG 30, Ghana

**Keywords:** reniform, nematode, population, ITS1, sequence, variants, male, female, Alabama

## Abstract

Around 300 different plant species are infected by the plant-parasitic reniform nematode (*Rotylenchulus reniformis*), including cotton. This is a devasting nematode with a preference for cotton; it is commonly found in Alabama farms and causes severe reduction in yields. Its first internal transcribed spacer (ITS1) region can be sequenced, and potential mutations can be found in order to study the population dynamics of the reniform nematode. The goal of our study was to sequence the ITS1 rDNA region in male and female RNs that were collected from BelleMina, Hamilton, and Lamons locations in Alabama. After separating the single male and female RNs from the samples collected from the three selected listed sites above, the ITS1 region was amplified selectively using specific primers, and the resulting products were cloned and sequenced. Two distinct bands were observed after DNA amplification of male and female nematodes at 550 bp and 730 bp, respectively. The analysis of sequenced fragments among the three populations showed variation in average nucleotide frequencies of female and male RNs. Singletons within the female and male Hamilton populations ranged from 7.8% to 10%, and the variable sites ranged from 13.4% to 26%. However, female and male BelleMina populations had singletons ranging from 7.1% to 19.7% and variable regions in the range of 13.9% to 49.3%. The female and male Lamons populations had singletons ranging from 2.5% to 8.7% and variable regions in the range of 2.9% to 14.2%. Phylogenetic (neighbor-joining) analysis for the two ITS1 fragments (ITS-550 and ITS-730) showed relatively high intra-nematode variability. Different clone sequences from an individual nematode often had greater similarity with other nematodes than with their own sequences. RNA fold analysis of the ITS1 sequences revealed varied stem and loop structures, suggesting both conserved and variable regions in the variants identified from female and male RNs, thus underscoring the presence of significant intra- and inter-nematodal variation among RN populations in Alabama.

## 1. Introduction

Plant-parasitic nematodes (PPNs) cause significant crop damage, accounting for approximately 10% (~USD 150 billion/year) of yield loss globally [1,2,3]. The reniform nematode (RN) is one of the top ten PPNs found in the United States (US); it belongs to the phylum Nematoda, order Tylenchida, family Hoplolaimidae, and genus Rotylenchulus [4]. RN is ranked as the seventh major nematode pest and has a broad host range, affecting approximately 350 plant species [5,6]. RN is a sedentary, microscopic, polyphagous, obligatory, highly adapted, semi-endoparasite that infects many crops and trees in almost all geographical locations, including temperate, subtropical, and tropical climates [7,8]. Known for their eclectic nature, RNs can be found in 70 nations worldwide, including the continents Africa, Asia, the Americas (North and South), Australia, and Europe [9,10,11]. The genus *Rotylenchulus* comprises ten confirmed species: *R. anamictus*, *R. clavicaudatus*, *R. leptus*, *R. macrodoratus*, *R. macrosoma (R. borealis*, a synonym), *R. macrosomoides*, *R. parvus*, *R. reniformis*, *R. sacchari*, and *R. variabilis* [4,12,13,14]. Among these, *R. reniformis* is the major parasitic species affecting economically important crops with preferential feeding on cotton (Gossypium) roots [9]. Also, *R. parvus* is the second most pathogenic form, while the remaining species in the genus are limited and have less economic importance.

As RN is highly adaptable, it can acclimate to global climate change by shortening its life cycle, expanding its distribution to new geographical territories, and converting non-infested fields to infested ones [5,9,15,16]. Over the last decade, RN has become a preponderating PPN in upland cotton in the southern US, including the states Alabama, Arkansas, Georgia, Louisiana, Mississippi, North Carolina, South Carolina, and Texas [17]. Crop type, soil type, and soil temperature are critical factors for RNs’ dispersion. The ideal RN survival temperature ranges between 25 °C and 29.5 °C [18]. It prefers fine-textured soils due to its habitat and edaphic preferences [19]. Differences in the RNs’ pathogenicity and reproduction on soybean and cotton crops cultivated in Louisiana, US, have been reported [20]. The life cycle of RN males and females includes egg, juvenile, and adult stages [21]. However, the female RN is infectious and forms a specialized feeding structure within the roots known as syncytium. Morphologically, the female RN is needle-shaped, whereas the male RN is curved with a spicule. The immature female has a slender and C-shaped body, while the mature one has a swollen and kidney-shaped body named Reniform. The morphological identification of RNs from mixed populations is elusive because there are several species within the genus and different types of races within the species.

Ribosomal RNA (rRNA) genes are commonly used in the identification of evolutionary relatedness among species or organisms [22]. The eukaryotic cistron of ribosomal DNA contains the first internal transcribed spacer (ITS1) between the genes encoding 18S and 5.8S rRNA. The ITS1 and ITS2 regions have been widely used in phylogenetic analysis [23]. Due to its non-coding structure, this spacer region exhibits a higher evolutionary rate. It is a marker for phylogenetic investigations of closely related animals, plants, and fungal taxa at the population and species levels. The identification of genetic diversity among the molds from *Ragi tape* in Java Island was performed using the ITS1/ITS2 ribosomal regions as molecular markers. This study confirmed that these molds were genetically diverse and not clustered with *Amylomyces rouxii* [24]. ITS markers were commonly used at the species level, while their usage as markers at the population level was limited. Recently, in antipatharians from shallow-water black corals, it was first confirmed that ITS rRNA regions can be used as markers at both the species and population levels [25]. The utilization of the ITS1 region as a marker for the identification of nematode species or populations has great potential in nematology [26]. Molecular techniques based on the restriction digestion of the ITS1 region were used to examine the species or populations of geographically dispersed nematodes [27]. Established phylogenetic relationships have been reconstructed by analyzing ITS1 rDNA sequences [28]. The phylogenetic analysis of ITS1 rDNA of the foliar nematode (*Aphelenchoides fragariae*) from Indonesia was found to be a genetically identical species to the *A. fragariae* spp. from other countries [29]. Recently, the molecular identification of neglected PPNs using 18S rDNA, ITS1/ITS2, and 28S in *Heterodera avenae*, *Xiphinema americanum*, *Ditylenchus dipsaci*, *Ditylenchus weischeri*, and *Punctodera stonei* have been reported [1,27,30,31].

Typically, rDNA regions were known to be highly conserved with little to no variations within or between the species, including higher taxa. However, evolutionary patterns in these conserved regions were observed across several species. In Paraplagusia japonica, the coexistence of three variants of ITS1-5.8S-ITS2, i.e., Types A, B, and C among intra- and inter-individuals, was reported, suggesting a non-conserved evolutionary pattern in the ITS1-5.8S-ITS2 rDNA region [32]. Higher inter and intra-specific variability levels were observed within the rDNA genes of the *Rotylenchulus* genus [9,33]. Types A and B for ITS1 [9,34,35] and 28S [33] for rDNA genes have been identified in RN. Two distinct ITS1 sequence lengths (549.3 bp and 720.8 bp) were determined in RN, collected from mixed Alabama populations. Female and male clone sequences were clustered regardless of sex and isolate used [28]. From these findings, it was inferred that the ITS1 rRNA region has both inter and intra-individual diversity in the RN. Similarly, two different ITS1 sequence variations were identified in the Japanese RN genome and found in both parthenogenetic and amphimictic populations [35]. Comparable findings have been reported from a study on lithoid and paranoid species. Short and varied length ITS1 regions were identified in seven lithoid and paranoid species, respectively, supporting the “hermit-to-king” crab hypothesis [36]. The molecular characterization of PPN population densities in crop fields is essential to guide farmers in improving the current nematode management practices.

To better understand the unexplored genetic variability in nematode populations, neglected geographical areas also must be studied at the molecular level using common genetic markers such as ITS1 rDNA. The current study aims to identify genetic diversity within and between ten individual male and female RNs collected from three underexplored locations of Alabama by sequencing the 18S ITS1 rDNA region. Further, the data generated in this study were compared to two known outgroups and a previous study that analyzed four amphimictic and four parthenogenetic populations.

## 2. Results

**Figure 1:**
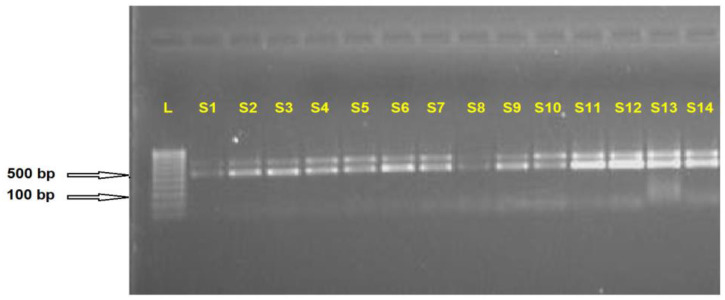
The ITS1 forward and reverse primers amplified the ITS1 rRNA regions of fourteen male reniform nematodes (S1–S14). The 100 bp DNA ladder ruler (L) confirmed two consensus bands identified.

In this study, the ITS1 rDNA region of the female and male reniform nematodes (RNs) from three different locations in Alabama (Hamilton, BelleMina, and Lamons) were sequenced, aligned (MSA), consensus-generated, and analyzed to determine their nucleotide frequencies, variable sites, phylogenetic relationships, and secondary structures. Among the three populations investigated, the amplification of the ITS1 region generated shorter (~550 bp) and longer (~730 bp) fragments for both males and females in the Hamilton and BelleMina RN populations (Figure 1). In contrast, in the Lamons population, both these fragments (~550 bp and ~730 bp) were found in females and a single band (~550 bp) was identified in males. However, the lengths of the individual longer and shorter ITS1 sequences identified in both reproductive types from the three locations varied significantly.

A total of 114 sequences were generated from the male and female Hamilton populations. Of these, an MSA analysis was conducted using 30 and 7 amplified ITS1 products of the female Hamilton population and we identified two consensus lengths of 557 bp (ITS1-557) and 732 bp (ITS1-732), respectively. Similarly, using 54 and 23 sequences as an input for the MSA analysis, we found two consensus lengths of 567 bp (ITS1-567) and 736 bp (ITS1-736), respectively, in the ITS1 region of the male Hamilton population. The average number of nucleotides of consensus products ITS1-557, ITS1-732, ITS1-567, and ITS1-736 identified in females and males were 546.6 bp, 724.7 bp, 549.8 bp, and 722.2 bp, respectively. In total, 40 sequences were generated from the male and female BelleMina populations. In the female RNs of the BelleMina population, 10 and 10 amplified ITS1 products were used in the MSA analysis to determine two consensus lengths, 588 bp (ITS1-588) and 735 bp (ITS1-735), respectively. Likewise, we discovered two consensus lengths of 570 bp (ITS1-570) and 729 bp (ITS1-729) by using ten sequences as input for the MSA analysis of short and long ITS1 regions from the male BelleMina population. Consensus products ITS1-588, ITS1-735, ITS1-570, and ITS1-729 obtained from BelleMina had average nucleotide counts of 529.9 bp, 715.8 bp, 550 bp, and 722.8 bp for males and females, respectively. Similarly, a total of 21 sequences were generated from the male and female Lamons populations. Of these, 15 sequences were used as input in the MSA analysis to determine two consensus lengths, 559 bp (ITS1-559) and 726 bp (ITS1-726), respectively. In contrast, only one consensus product of 559 bp (ITS1-559) was discovered in the ITS1 region of the male Lamons population when utilizing six sequences as input in the MSA analysis. The average number of nucleotides in 549.2 bp and 714.8 bp consensus products ITS1-559 and ITS1-726 was discovered in the female population, while only a short (ITS1-559) consensus fragment with an average length of 549.7 bp was identified in the male population.

**Figure 2:**
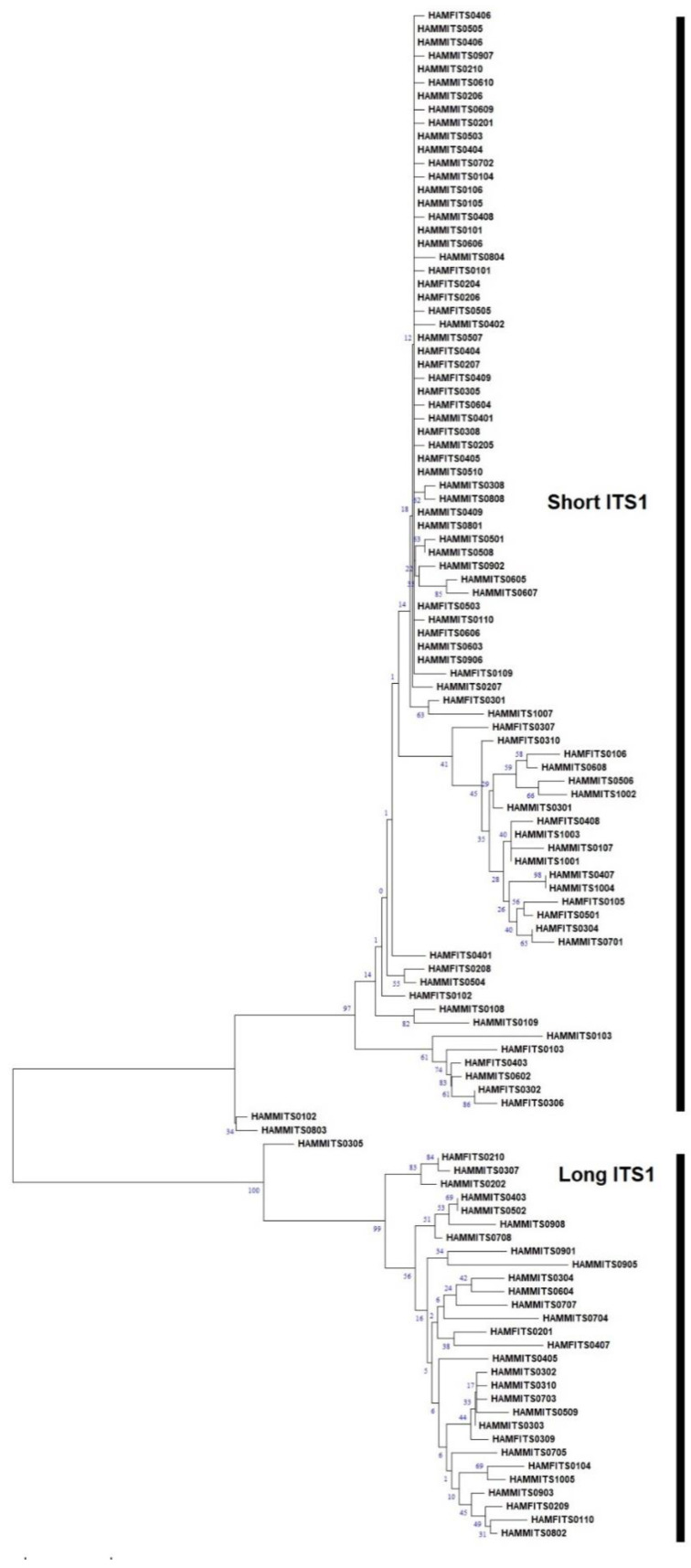
Neighbor-joining (NJ) analysis was used to create a phylogram for 84 mixed male and female reniform nematode ITS1 clones for shorter (ITS-567) and 30 mixed female and male reniform nematode ITS1 clones for longer (ITS-736) fragments from the Hamilton population. The percentages of bootstrap replicates that support groups A and B and subgroup clades are displayed at the branch points. Figure Legend: HAM: Hamilton, F: Female, M: Male, ITS: ITS1. The four-digit number at the end denotes the clone number.

**Figure 3:**
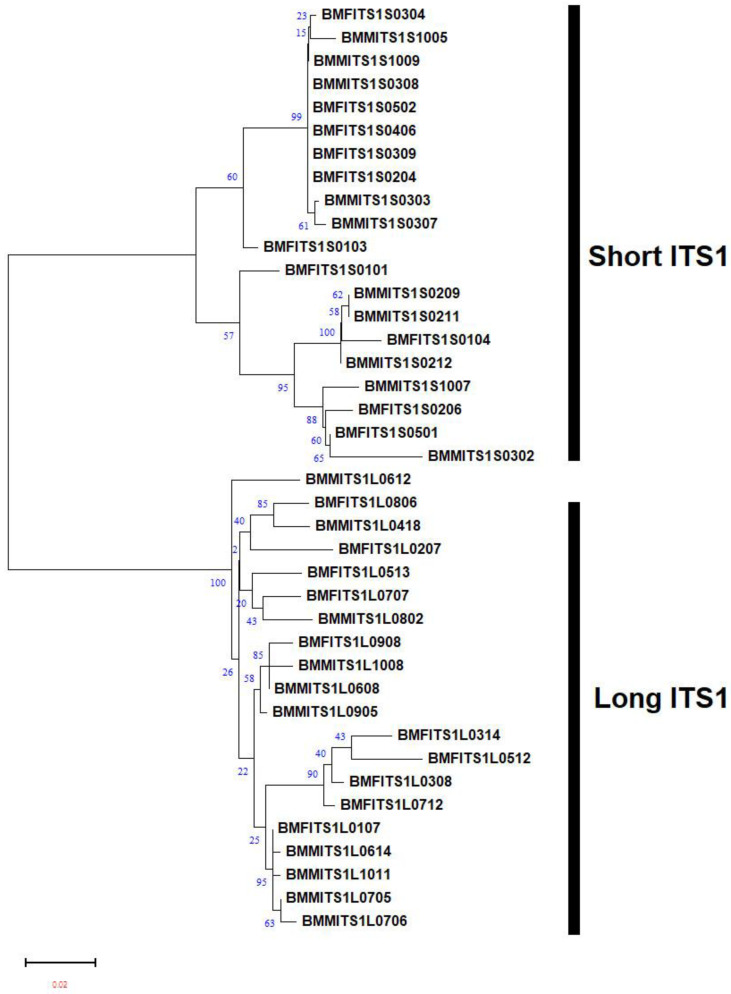
Neighbor-joining (NJ) analysis was used to create a phylogram for 20 mixed male and female reniform nematode ITS1 clones for shorter (ITS-588) and 20 mixed female and male reniform nematode ITS1 clones for longer (ITS-734) fragments from the BelleMina population. The percentages of bootstrap replicates that support groups A and B and subgroup clades are displayed at the branch points. Figure Legend: BM: BelleMina, F: Female, M: Male, ITS: ITS1. The four-digit number at the end denotes the clone number.

**Figure 4:**
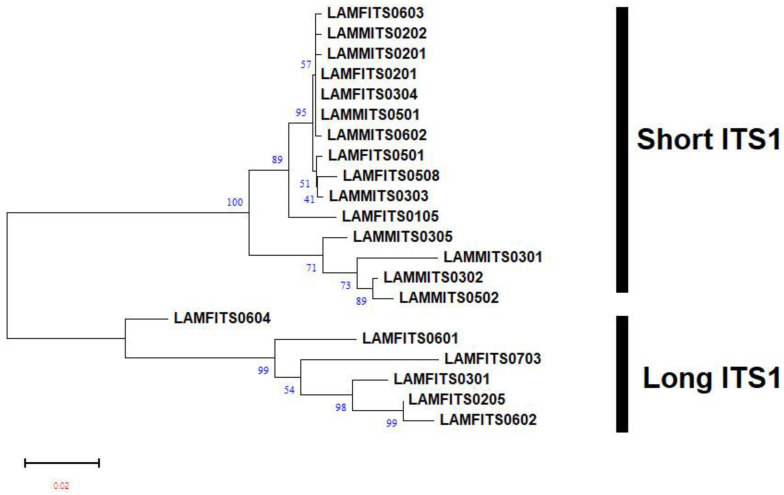
Neighbor-joining (NJ) analysis was used to create a phylogram for 15 mixed male and female reniform nematode ITS1 clones for shorter (ITS-559) and 6 female reniform nematode ITS1 clones for longer (ITS-726) fragments from the Lamons population. The percentages of bootstrap replicates that support groups A and B and subgroup clades are displayed at the branch points. Figure Legend: LAM: Lamons, F: Female, M: Male, ITS: ITS1. The four-digit number at the end denotes the clone number.

The nucleotide frequencies of consensus length products identified in both female and male RNs from the three populations are presented (Table 1). Based on the frequencies observed, it can be inferred that the occurrence of T/C/A/G varied significantly in the two products identified from female and male RNs. The average nucleotide frequencies of T, C, A, and G were ~27%, ~24%, ~24%, and ~23% for shorter fragment (ITS1-557/ITS1-567) and ~25%, ~25%, ~22%, and ~26% for longer fragment (ITS1-732/ITS1-736), respectively. This shows the relatively high occurrence of Ts in shorter and Gs in the longer fragments identified in both the male and female populations of Hamilton. A similar trend was also observed in both the male and female populations of BelleMina and in the female population of Lamons. Four regions, namely, singletons, conserved, parsimony-informative, and variable sites, were identified within the aligned sequences of two consensus lengths. For ITS1-567, these sites involved singletons (57/567), conserved sites (418/567), parsimony-informative sites (90/567), and variable sites (147/567). Similarly, for ITS1-736, the sites identified were singletons (72/736), conserved sites (563/736), parsimony-informative sites (98/736), and variable sites (170/736) (Table 2). The NJ tree analysis for two consensus product lengths (~500 bp and ~700 bp) identified in the female and male RNs from Hamilton (Figure 2), BelleMina (Figure 3), and Lamons (Figure 4) populations discussed above showed the existence of A and B groups and 3–4 subgroups within each group. Inter- and intra-nematodal variation was observed among these clones. These subgroupings were mostly made up of a combination of female and male clones. However, there were instances where only female or male clones clustered together in a subgroup.

**Figure 5:**
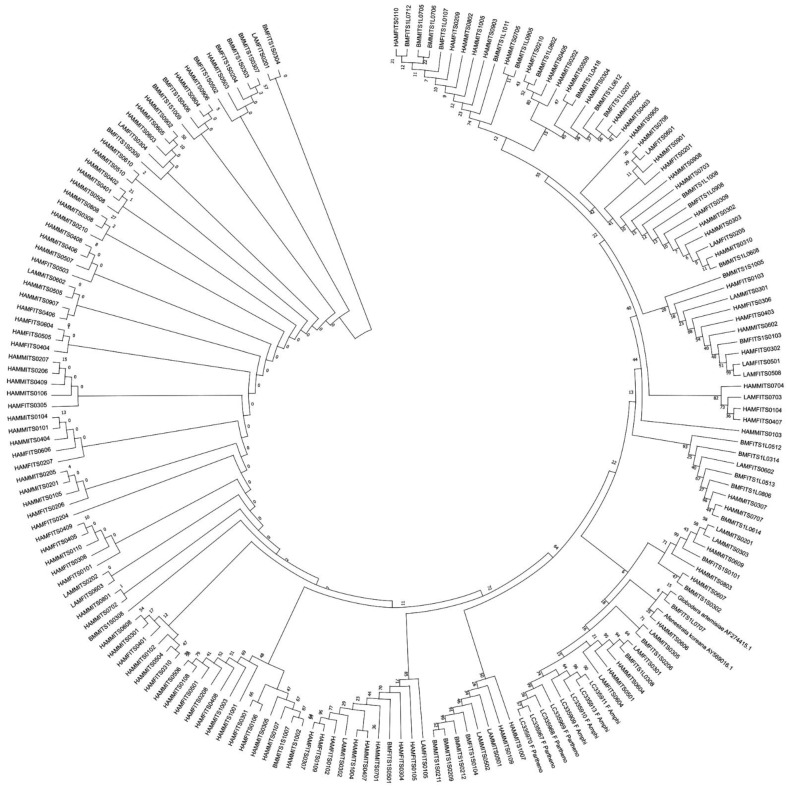
Female and male ITS1 clones from the three populations were compared to two outgroups and four amphimictic and four parthenogenetic strains of reniform nematode sequences using a circular phylogram produced using neighbor-joining (NJ) analysis. The numbers at each branch point represent the bootstrap values (10,000 replicates). The clone ID (or) sample ID (or) accession number is displayed in the outer ring.

**Figure 6:**
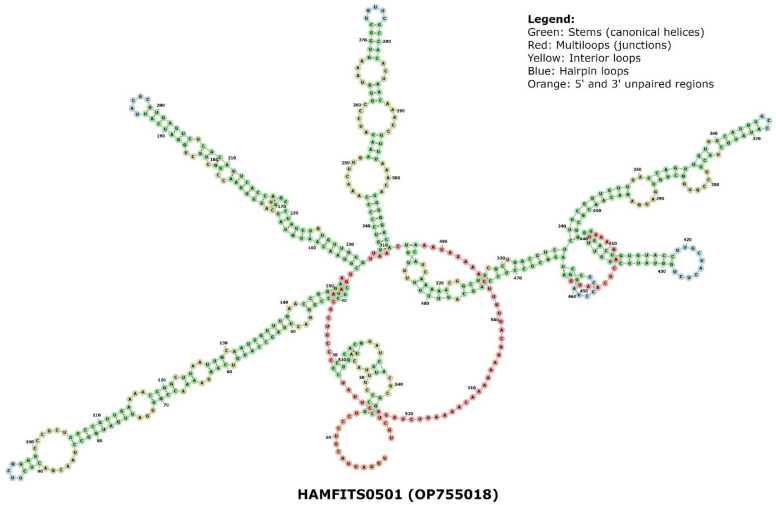
RNA fold (FORNA) structure of a representative ITS1 sequence randomly selected from the Hamilton population (e.g., HAMFITS0501).

**Figure 7:**
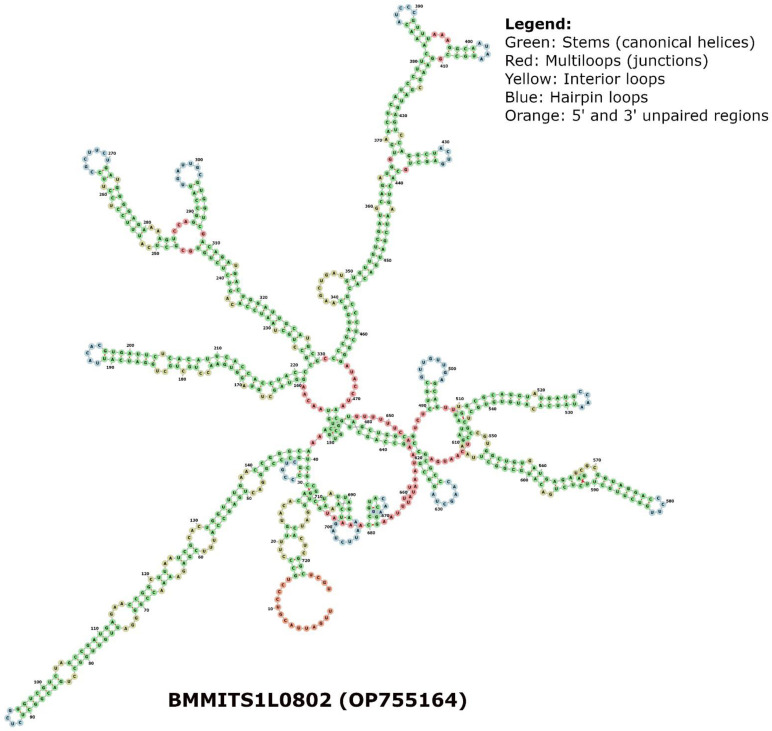
RNA fold (FORNA) structure of a representative ITS1 sequence randomly selected from the BelleMina population (e.g., BMMITS1L0802).

**Figure 8:**
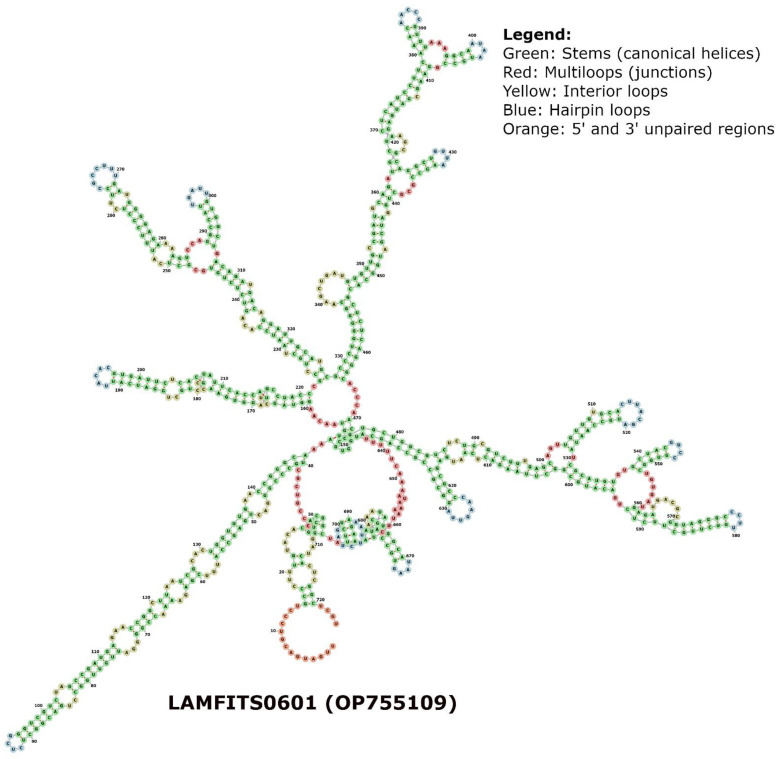
RNA fold (FORNA) structure of a representative ITS1 sequence randomly selected from the Lamons population (e.g., LAMFITS0601).

Additionally, we performed a combined phylogram analysis using the NJ method with 175 sequences from this study, ITS1 sequences from two known PPNs (outgroups), and four amphimictic and four parthenogenetic sequences from a previous study [35] to reveal the relatedness of long and short sequences of the males and females identified in the three locations. Our combined circular phylogram with RN clone sequences from Hamilton, BelleMina, and Lamons showed that the sequence length variants formed different clusters. In contrast, the remaining ITS1 sequences were mixed with two reproductive types and outgroups rather than forming distinct clusters. Meanwhile, the outgroup sequences (*Afenestrata koreana* and *Globodera artemisiae*), and amphimictic, and parthenogenetic RN sequences were closely clustered (Figure 5). Phylogenetic analysis using rRNA secondary structure prediction algorithms has evolutionary significance as it relies largely on canonical and non-canonical base pairing rules and often forms helix-, stem-, bulge-, and loop-like structures. In order to consider an algorithm efficient in secondary structure prediction, it must have an energy minimization approach that considers all possible thermodynamic properties. In nematodes, the secondary structural patterns in rRNA are evolutionarily conserved, and the differences in these structures can be used as a structural marker. The minimum (Gibbs) free energy (MFE, ΔG) is an important parameter in predicting a secondary structure of ITS1 rDNA, and it varies from −40 to −100 kcal/mol in various RN species. The ΔG required to build the secondary structure of different ITS sequences in RN varied from −54 to −108 kcal/mol for core ITS1 regions. The folding arrangement of the ITS1 secondary structure in the eukaryotic taxa that have been studied thus far varies among taxonomic groups, but the area is distinguished by the presence of a large central loop and several double-stranded helices. Among the three RN species investigated, the MFE secondary structures based on the ITS1 region displayed the same consistent folding pattern of a core loop with a varying number of helices as observed in other eukaryotes. The RNA fold analysis of ITS1 sequences randomly selected from the Hamilton, BelleMina, and Lamons populations were presented (Figure 6, Figure 7 and Figure 8).

## 3. Discussion

Studies conducted on various species have reported variations within the ITS1 rDNA region. The underlying reasons for such variations include alterations in nucleotide frequencies, mutations, the presence of short and long repeats, and microsatellites [37,38,39,40]. The current study discusses potential reasons for ITS1 rDNA variations in RN populations collected from three cotton-growing locations in Alabama in the United States. It then compares their relatedness with two outgroup genera and four amphimictic, and four parthenogenic RN populations. Significant intra-individual variations were observed in all three RN populations studied, and their occurrence within the nematode genomes was likely due to the existence of more than one copy of ITS1. These copies were generated due to nucleotide changes and varying numbers of short, repetitive motifs within the sequence. The presence of two or three types (shorter or longer) of ITS1 sequences has been reported in several evolutionarily lower animal and parasitic species [38,39,40]. A study on coral populations examined inter- and intra-specific variability in the ITS regions of rDNA. They demonstrated that the amplified rDNA fragment lengths varied significantly among coral species, including *Stylophora pistillata* (850 bp), *Seriatopora hystrix* (790 bp), *Goniopora tenuidens* (810 bp), *Porites lobata* (760 bp), *Heliofungia actiniformis* (740 bp), *Acropora valida* (490 bp), and *Acropora longicyathus* (490 bp). The intraspecific variability in ITS1 sequences that they identified in coral species was primarily due to insertions/deletions or base substitutions, i.e., 15% in *G. tenuidens*, 2% in *H. actiniformis*, 11% in *A. longicyathus*, and 31% in *S. pistillata* [41]. Studies conducted in tonguefish species *Cynoglossus melampetalus* [23] and *Paraplagus japonica* [32] showed high intra-individual variation within the ITS1-5.8S-ITS2 rDNA gene. One of these studies identified three divergent type (A, B, and C) sequences that varied in length, GC content, secondary structure stability, and lowest free energy. Similarly, the evolutionarily inferior tapeworm, *Atractolytocestus huronensis*, a parasite found in carps in the Huron River (Michigan, USA), showed intra-individual ITS sequence variations due to its triploid nature and the presence of several rDNA loci [42]. In a study conducted on amphistomes or flukes, variations in ITS1 were observed. Two ITS1 length variants were identified in the flukes based on the number of five-nucleotide repeats. These variations were possibly due to high mutation rates and repeat regions [43]. Likewise, differences in ITS1 sequence lengths were observed in rumen flukes (*Paramphistomum cervi*) of Pakistani buffaloes, and eight transversions were identified as possible reasons for the intra-individual variation [44].

Variations in nematode ITS evolution rates are probably responsible for the higher intra-nematode divergence that is often found in the ITS1 genes [45]. A study on 20 reniform nematode (RN) populations obtained from countries including the US, Brazil, Colombia, Honduras, and Japan identified ITS1 sequence divergence. The consensus sequence length of the amplified products was 348 bp. The phylogenetic analysis of ITS1 amplicons revealed no polymorphisms in amphimictic populations. On the other hand, a parthenogenic population from Japan showed 11.78% (41/348 bp) ITS1 sequence divergence [34]. Contrarily, a limited variation in the ITS1 region and a possible existence of conserved ITS1 paralog in these RNs have been reported [46]. In a different study, using three populations of *R. reniformis*, 60 sequences were analyzed to identify two rDNA variant types in the ITS, RN_VAR1 (1250 bp) and RN_VAR2 (1400 bp). Using multiple sequence alignment analysis, the percent identity, sequence length, and GC content of RN_VAR1 were 89.9–100%, 705–712 bp, and 45.1–46.7%, respectively. The percent identity, sequence length, and GC content of RN_VAR2 were 91.4–99.8%, 854–860 bp, and 48.4–50.0%, respectively. Phylogenetic analyses using the NJ approach revealed that both sequence length variants formed different clusters. However, a few mixed clusters were identified between the shorter and longer fragments due to intra-individual variation [47]. Similarly, our study identified variations in the ITS1 sequences between the female and male RN clones. The female and male Hamilton, BelleMina, and Lamons populations had 2.2%, 3.4%, and 1.4% variation in the short ITS1 fragment (ITS-550). The highest and lowest mutation rates observed in the female BelleMina and male Lamons clones were 19.7% and 3.9%, respectively. However, for the large ITS1 fragment (ITS-720), 2% and 3% differences were observed in Hamilton and BelleMina female and male populations, respectively. The highest and lowest mutation rates for ITS-720 were 10.1% and 7.1% for female and male BelleMina clones, respectively. For both female and male clones, the average nucleotide frequencies in the ITS-550 for the three populations of RNs were in the descending order of Ts > Cs > As > Gs. Similarly, for both female and male RN clones, the nucleotide frequencies for the ITS1 fragment (ITS-730) were in the decreasing order of Gs > Cs > Ts > As. In a previous study, average nucleotide frequencies in the short ITS1 (ITS1S) for T, C, A, and G were 27.1%, 24.7%, 24.4%, and 23.9%, respectively, for RN isolates that were sequenced from mixed populations in Alabama. However, the average G, C, T, and A nucleotide frequencies for the larger ITS fragments (ITS1L) were 26.2%, 25.9%, 25.7%, and 22.2%, respectively [48]. The GC content identified in our study varied between 47% and 52%, as reported previously [47,48]. Comparing the nucleotide frequencies identified in this study against the two studies listed above revealed similar nucleotide percentages for ITS1 segments (Table 1).

A comprehensive study included six *Rotylenchulus* species, *R. clavicaudatus*, *R. leptus*, *R. macrodoratus*, *R. macrosoma*, *R. reniformis* and *R. sacchari* from South Africa, USA, Italy, and Spain to characterize the molecular diversity of ITS rRNA, D2-D3 of 28S rRNA, cytochrome c oxidase subunit, *coxI* mtDNA and hsp90 gene sequences. Of these, the genomes of *R. reniformis* and *R. macrosomoides* disclosed two different rRNA gene types, while the genome of *R. macrosoma* revealed three different rRNA gene types. The variability in these gene types was used to develop species-specific primers that helped perform the rapid PCR diagnostics of *R. reniformis* [33]. A study that included six nations and six host species from the Mediterranean region was conducted to determine the molecular diversity in *R. macrodoratus* and *R. macrosoma*. In their genomes, two different 28S rRNA gene types were identified in *R. macrosoma* and *R. macrodoratus*, while two ITS types were identified only in *R. macrodoratus*. Phylogenetic analyses of ITS gene types of *R. macrosoma* samples collected from Greece and Spain were clustered distinctly [9]. In a study, the genetic diversity of 16 populations of *R. macrosoma* from Europe and 12 populations of *R. borealis* from the Netherlands were compared with available nematode collections. The synonym of *R. macrosoma* with *R. borealis* was established by the phylogenetic analysis of molecular markers utilizing *coxI* mtDNA and nuclear rDNA (28S, ITS1) genes. In addition, the genome of *R. borealis* (=*macrosoma*) contains two unique rRNA genes, namely D2–D3 (A and B types) [10]. In our study, comparing Hamilton, BelleMina, and Lamons populations, we identified A and B groups and 2–3 subgroups within each group. A study was conducted with *R. macrosoma* samples collected across eight countries from Europe with four host species to characterize RN molecular diversity. Significant amounts of genetic diversity and structural variability were identified in *R. macrosoma* populations by phylogenetic analysis with *coxI*, 28S, and ITS1 markers [11].

In Japan, a molecular study was carried out on two distinct reproductive types (amphimictic and parthenogenic) of *R. reniformis* to elucidate the relationships and their pathogenicity based on variations of ITS of ribosomal sequences and the mtDNA *coxI* gene. They found long and short rDNA ITS sequences from the amplicons of individual nematodes. Phylogenetic analysis using the NJ method revealed that amphimictic and parthenogenetic types formed distinct clusters of long or short ITSs, except for two amplicons. Within these clusters, most amphimictic and parthenogenetic populations were grouped separately. Further, the mtDNA *coxI* gene confirmed the phylogenetic relationships between the reproductive types [35]. In our study, the ITS1 gene amplification revealed two fragments (ITS-550 and ITS-730) with an average consensus length of 550 and 720 bp. Similarly, two ITS1 sequences (short and long) with lengths of 809 bp and 947–976 bp were identified in RNs in Japan [35]. The lengths of these sequences identified previously [35,47] were significantly greater than those of the two ITS1 segments identified in our study, possibly due to fewer repeat regions in the investigated RN populations. Significant intra-individual diversity was seen in the ITS fragment of RN using the NJ method (ITS-550 and ITS-730); different cloned sequences from one nematode typically showed more significant similarities with other nematodes than with their sequences. Internal repeats and intra-nematode variations were observed among the female and male clones. Our combined circular phylogram analysis revealed that 175 RN clones from three locations were clustered distinctly based on sequence length, but the Hamilton, BelleMina, and Lamons populations were mixed with the two outgroup species (*Afenestrata koreana* and *Globodera artemisiae*) and four amphimictic, and four parthenogenetic populations from a previous study [35]. Further, the amphimictic and parthenogenetic populations were closely clustered compared to our RN clones.

Combining molecular and structural characteristics for nematode phylogeny analysis will aid in building reliable and accurate secondary-structure models based on the consensus ITS sequences [49]. Multiple sequence alignment analysis followed by phylogenetic analyses using structural information is an ideal approach for accurately demonstrating relationships between clades and most likely avoiding misinterpretations while analyzing monophyletic or paraphyletic groups [50]. Alteration in nematode ITS gene evolution rates is probably responsible for the large amounts of intra-nematode variations found in the ITS1 genes, and often, these changes are associated with secondary structures such as stems, loops, and helices [45]. Our secondary structure analysis of three ITS1 rDNA sequences included a representative sequence from each of three locations in Alabama. These analyses identified a main central loop (I) and four (II–V) to six (II–VII) double-stranded helices with two to three subloops and over 70% of the nucleotides involved in base pairing (Figure 6, Figure 7 and Figure 8). Most of these base pairings identified were canonical (A–U/C–G), and a few were non-canonical (G–U/U–U/A–G/C–U) base pairings. However, the overall sequence lengths and number of nucleotides within the helices varied slightly. Similar patterns in sequence alignments and secondary structure predictions have been reported in nematode species, i.e., Hoplolaimids [40,49,50,51]. Among the 175 RN sequences used in this study, the most abundant DNA repeats identified were dinucleotides (GT, AC, CA, and GA), trinucleotides (CTG, GCT, ACA, and GTG), and tetranucleotides (TTTT and AAAA) that were similar to the previous findings [52]. Of the 175 RN clones, 83 had the longest DNA repeat motif with 22 bp (GTTGTTGCATTGCTAATGTGCT), which could be an essential structural element.

## 4. Materials and Methods

### 4.1. Nematode DNA Extractions from Soil

Three locations in Alabama infested with reniform nematodes (RN) were selected to collect samples, including Hamilton and Lamon farms (Lamons) in Lawerence and the BelleMina site in Limestone. These regions were selected based on our previous greenhouse study, which revealed three categories using canonical discriminant analysis (CDA) of thirteen morphometric features tested on nine and one infected population of RNs from Alabama and Mississippi, respectively [48]. Initially, RNs of both sexes were separated from the soil samples. Then, DNA was extracted from ten female and ten male worms using a DNeasy blood and tissue kit (Qiagen, Inc., Valencia, CA, USA) per the manufacturer’s instructions.

### 4.2. Quality Check, PCR Amplification, and Determination of the Product

A Nanodrop 2000 spectrophotometer was utilized to assess the quality and quantity of isolated gDNA. The 260/280 and 260/230 ratios ranged from 1.8 to 2.0 for each sample and were used to evaluate the sample quality. In the PCR reaction, 250 µL of template DNA (1.0 ng/µL), 2.5 µL of PCR buffer (10X), 1.0 µL of MgCl_2_ (50 mM), 0.5 µL of dNTPs (10 mM), 0.5 µL of forward (RN_ITS1_F, 5′-TTGATTACGTCCCTGCCCTTT-3′) and reverse (RN_ITS1_R, 5′-ACGAGCCGAGTGATCCACCG-3′) primers (10 µm), 0.2 µL of platinum Taq, and 17.3 µL of sterile, DNase-free water were used. The PCR conditions used on a tetrad (Bio-Rad, Hercules, CA, USA) thermal cycler were 94 °C for two minutes, thirty cycles of 94 °C for thirty seconds, 60 °C for thirty seconds, and 68 °C for one minute, and a final extension step for seven minutes at 72 °C. The size of the amplified products was measured using a 100 bp DNA ruler on a 1% agarose gel.

### 4.3. Cloning and Colony PCR

Ten male and female individual nematodes ITS1 region was amplified, and the amplicons were purified before cloning using a QIAquick PCR Purification Kit (Qiagen, Inc.) in accordance with the manufacturer’s instructions. The purified fragments were cloned into a plasmid vector using the TOPO TA Cloning Kit (Invitrogen, Carlsbad, CA, USA). The ligation reaction mixture comprised 4 μL of pure DNA fragment, 1 μL of TOPO vector, and 1.2 M NaCl and 0.06 M MgCl_2_ salt solution. The ligated product was transformed, and the transformants were screened using colony PCR. Following five minutes at 94 °C, there are 40 cycles of 94 °C for thirty seconds, 55 °C for one minute, and 72 °C for one minute in the PCR reaction. The last extension phase lasted 10 min at 72 °C. After screening and confirmation using colony PCR, the selected bacterial colonies were grown in 1.3 mL of liquid Luria-Bertani (LB) media with 100 μg/mL ampicillin in 96-well blocks using an Innova 4300 rotary incubator shaker. For 24 h, the cells were shaken at 300 rpm and 37 °C. The cells were extracted using an Eppendorf 5804R centrifuge at 2000× *g* for 12 min. Using the QIAprep Spin Mini-prep kit, plasmid DNA was extracted from the grown cells in accordance with the manufacturer’s suggested methodology (Qiagen, Inc.).

### 4.4. Sequencing

Plasmid inserts were sequenced on the ABI 3730 nucleotide sequencer using the Applied Biosystems (Foster City, CA, USA) ABI PRISM Big Dye Terminator cycle sequencing ready reaction kit. The ITS1 genes of the RN were sequenced using primers T7 (5′-TAATACGACTCACTATAGGG-3′) and T3 (5′-TAACCCTCACTAAAGGGA-3′). Using the standard nucleotide-nucleotide BLAST [blastn] on the NCBI website (http://www.ncbi.nlm.nih.gov/Blast.cgi (accessed on 20 June 2022), the plasmid insert sequences were aligned to nematode sequences.

### 4.5. Alignment and Phylogenetic Analysis

After aligning the sequenced products with the use of the SeqMan Pro tool in the DNASTAR Lasergene v8.0 program (DNASTAR Inc., Madison, WI, USA), consensus sequences were produced. Sequences that were not a part of the target amplification products were eliminated. The ITS1 sequences, which contained both male and female clones, underwent multiple sequence alignment (MSA) and phylogenetic analysis using the Molecular Evolutionary Genetics Analysis (MEGA) software version 11.0. Initially, alignment (MSA) was done with ClustalW using default parameters (gap opening = 15.00; gap extension = 6.66). Then, with the aligned sequences, phylogenetic trees were reconstructed by the Neighbor-joining (NJ) method with 10,000 replications of the bootstrap test of phylogeny using a 64,238 random seed. The nucleotide model was chosen based on the following criteria: maximum/greatest composite likelihood, substitutions (d: transitions + transversions), consistent rates among sites, homogenous pattern among lineages, and gaps/missing data treated as a complete deletion. Moreover, two additional sequences from *Afenestrata koreana* (AY569016) and *Globodera artemisiae* (AF274415) were used as outgroups. Recently reported RN’s ITS1 sequences from four amphimictic (LC335909, LC335910, LC335911, LC335913) and four parthenogenetic (LC335967, LC335968, LC335969, LC335970) populations [35] were downloaded and used in our phylogenetic analysis. Less than 50% of bootstrap replicates of a partition’s related branches collapsed. The Maximum Composite Likelihood method calculated the evolutionary distances expressed in base substitutions per site [53]. This analysis employed a total of 175 nucleotide sequences. The pairwise deletion feature removed the anonymous nucleotides from the sequences, leaving 1365 locations in the final dataset. Further, the features extracted from MSA generated here were used to identify conserved (C), variable sites (V), Parsimony-informative sites (Pi), and Singletons (S). The GenBank accession numbers for the ITS1 sequences of the RN populations from the Hamilton, Lamons, and BelleMina sites are OP754993–OP755106, OP755107–OP755127, and OP755128–OP755167, respectively.

Using the RNAfold function of the Vienna RNA package, the minimal free energy (MFE) secondary structure of a single ITS RNA sequence was predicted [54,55]. Apart from MFE folding, the partition function (PF) approach [56] was utilized to compute equilibrium base-pairing probabilities. Due to the error-prone nature of RNA structure prediction, these secondary structures were further supplemented and validated by the centroid structures [57]. As a result, the structure with the smallest base-pair distance to every other structure in the thermodynamic ensemble can be calculated (*p* > 0.5). The kinetics of nucleotide sequence hybridization are influenced by secondary structure. The sequences that show negative and positive Gibbs free energy (ΔG) respectively form stable and unstable structures during the secondary structure formation. A main central loop and multiple double-stranded helices often characterize these secondary structures. Three sample ITS sequences were chosen from different clades, and their MFE structures were used as reliable predictions based on the strong similarity between their centroid and helices. The secondary structures of representative ITS1 sequences randomly selected from the Hamilton (HAMFITS0501; OP755018), BelleMina (BMMITS1L0802; OP755164), and Lamons (LAMFITS0601; OP755109) population with sequence lengths 548 bp, 726 bp, and 724 bp, respectively were obtained from *R. reniformis* clade. Further, we compared these structures with five previously reported clades: *Scutellonema* sp. (JX472065), *Heterodera avenae* (KC736877), *Hoplolaimus stephanus* (HQ678736), *Afenestrata koreana* (AY569016), and *Helicotylenchus pseudorobustus* (KM506880).

## 5. Conclusions

Our research revealed high intra-individual variability within the ITS1 rDNA of male and female reniform nematodes (RN) from all three populations. Phylogenetic analysis identified two variants for the ITS1 rRNA gene in both male and female RNs. These findings contradict the hypothesis of concerted evolution, which states that numerous copies of rDNA should be homogenized within species to increase translation efficiency and reduce intra-specific variation. Moreover, the domains in the ITS1 rRNA gene of the RN might not be conserved, which could evolve quickly, thereby enhancing the intra-individual variability. The results of the present study show that RN variants were found in three Alabama locations; their occurrence is expanding from infested fields to non-infested ones, and the extent of damage they cause is increasing rapidly. Due to the dynamic nature of the RN and its level of infestation, this is an alarming issue that needs to be addressed. A more comprehensive sampling across Alabama is required to explicitly define the species spread or expansion and geographical location-specific genetic variability.

## Figures and Tables

**Table 1:** Nucleotide frequencies of ITS1 gene fragments identified from three locations in Alabama.

	Hamilton Population				BelleMina Population				Lamons Population			
	Female		Male		Female		Male		Female		Male	
	ITS1-557	ITS1-732	ITS1-567	ITS1-736	ITS1-588	ITS1-735	ITS1-570	ITS1-729	ITS1-559	ITS1-726	ITS1-559	ITS1-726
T%	27.20	25.70	27.20	25.70	27.10	25.70	27.10	25.70	27.40	25.70	27.10	NA
C%	24.60	25.90	24.60	25.60	24.50	26.10	24.60	25.70	24.40	25.80	24.80	NA
A%	24.30	22.10	24.40	22.30	24.40	22.30	24.50	22.10	24.30	22.20	24.40	NA
G%	23.80	26.40	23.80	26.30	24.10	25.90	23.80	26.50	23.90	26.40	23.80	NA

**Table 2:** Four different sites of ITS1 gene fragments identified from three locations in Alabama.

	Hamilton Population				BelleMina Population				Lamons Population			
	Female		Male		Female		Male		Female		Male	
	ITS1-557	ITS1-732	ITS1-567	ITS1-736	ITS1-588	ITS1-735	ITS1-570	ITS1-729	ITS1-559	ITS1-726	ITS1-559	ITS1-726
Singletons	44/557	57/732	57/567	72/736	116/588	74/735	93/570	52/729	14/559	63/726	22/559	NA
Conserved sites	462/557	631/732	418/567	563/736	275/588	580/735	385/570	626/729	534/559	621/726	503/559	NA
Parsimony-informative sites	51/557	41/732	90/567	98/736	172/588	74/735	85/570	49/729	2/559	40/726	29/559	NA
Variable sites	95/557	98/732	147/567	170/736	290/588	148/735	178/570	101/729	16/559	103/726	51/559	NA

## Data Availability

Data reported in this article is available under GenBank accessions between OP754993 and OP755167.
